# In Vitro Evaluation of Different Prebiotics on the Modulation of Gut Microbiota Composition and Function in Morbid Obese and Normal-Weight Subjects

**DOI:** 10.3390/ijms21030906

**Published:** 2020-01-30

**Authors:** Alicja M. Nogacka, Nuria Salazar, Silvia Arboleya, Patricia Ruas-Madiedo, Leonardo Mancabelli, Adolfo Suarez, Ceferino Martinez-Faedo, Marco Ventura, Takumi Tochio, Katsuaki Hirano, Akihito Endo, Clara G. de los Reyes-Gavilán, Miguel Gueimonde

**Affiliations:** 1 Department of Microbiology and Biochemistry of Dairy Products, Instituto de Productos Lácteos de Asturias (IPLA-CSIC), 33300 Villaviciosa, Asturias, Spain; nuriasg@ipla.csic.es (N.S.); silvia.arboleya@ipla.csic.es (S.A.); ruas-madiedo@ipla.csic.es (P.R.-M.); greyes_gavilan@ipla.csic.es (C.G.d.l.R.-G.); mgueimonde@ipla.csic.es (M.G.); 2 Diet, Human Microbiota and Health Group, Institute of Health Research of the Principality of Asturias (ISPA), 33011 Oviedo, Spain; adolfo.suarez@hcabuenes.es; 3 Functionality and Ecology of Beneficial Microorganisms, Institute of Health Research of the Principality of Asturias (ISPA), 33011 Oviedo, Spain; 4 Laboratory of Probiogenomics, Department of Life Sciences, University of Parma, 43121 Parma, Italy; leonardo.mancabelli@genprobio.com (L.M.); marco.ventura@unipr.it (M.V.); 5 Digestive Service, Central University Hospital of Asturias (HUCA), 33011 Oviedo, Asturias, Spain; 6 Endocrinology and Nutrition Service, Central University Hospital of Asturias (HUCA), 33011 Oviedo, Asturias, Spain; ceferinofaedo@gmail.com; 7 Endocrinology, Nutrition, Diabetes and Obesity Group, Institute of Health Research of the Principality of Asturias (ISPA), 33011 Oviedo, Spain; 8 β-Food Sciences Co., Chita 478-0046, Japan; t-tochio@bfsci.co.jp (T.T.); k-hirano@bfsci.co.jp (K.H.); 9 Department of Food and Cosmetic Science, Tokyo University of Agriculture, Abashiri 099-2493, Japan; a3endou@nodai.ac.jp

**Keywords:** in vitro model, microbiota, prebiotics, gas production, obesity, functionality, HT29, RTCA, SCFA, bifidobacterial-ITS

## Abstract

The gut microbiota remains relatively stable during adulthood; however, certain intrinsic and environmental factors can lead to microbiota dysbiosis. Its restoration towards a healthy condition using best-suited prebiotics requires previous development of in vitro models for evaluating their functionality. Herein, we carried out fecal cultures with microbiota from healthy normal-weight and morbid obese adults. Cultures were supplemented with different inulin-type fructans (1-kestose, Actilight, P95, Synergy1 and Inulin) and a galactooligosaccharide. Their impact on the gut microbiota was assessed by monitoring gas production and evaluating changes in the microbiota composition (qPCR and 16S rRNA gene profiling) and metabolic activity (gas chromatography). Additionally, the effect on the bifidobacterial species was assessed (ITS-sequencing). Moreover, the functionality of the microbiota before and after prebiotic-modulation was determined in an in vitro model of interaction with an intestinal cell line. In general, 1-kestose was the compound showing the largest effects. The modulation with prebiotics led to significant increases in the *Bacteroides* group and *Faecalibacterium* in obese subjects, whereas in normal-weight individuals, substantial rises in *Bifidobacterium* and *Faecalibacterium* were appreciated. Notably, the results obtained showed differences in the responses among the tested compounds but also among the studied human populations, indicating the need for developing population-specific products.

## 1. Introduction

The human intestinal microbiota represents a very complex and diverse microbial ecosystem that remains relatively stable during adult life [1]. However, several intrinsic and environmental factors can disrupt the microbiota composition, causing a microbiota “dysbiosis” [2]. Given the frequent association of dysbiosis with different disease states, the restoration of the microbiota through dietary modulation strategies could be a suitable approach. Among the different microbiota-modulation strategies, the administration of prebiotic supplements has been associated with health benefits to the gastrointestinal tract, cardiometabolism, mental health and mineral absorption, among others [3]. An international experts group has defined a prebiotic as a “substrate that is selectively utilized by host microorganisms conferring a health benefit” [3]. Most often, these substrates are complex carbohydrate moieties that, due to the presence of β-glycosidic bonds, are resistant to digestion during their passage through the gastrointestinal tract, reaching almost intact the large intestine, where they can be metabolized by the intestinal microbiota [4]. However, it is important to underline that resistance to digestion is not enough, and, by definition, prebiotics substrate must be selectively utilized by the microorganisms, with a subsequent promotion of health. 

Traditionally, the selective nature of prebiotics have been specifically associated with the genera *Bifidobacterium* and *Lactobacillus* [3]. Nevertheless, during the last two decades, the development of culture-independent technologies has demonstrated that other intestinal microorganisms could be affected as well. Among these, some butyrate producers, such as members of *Clostridium* cluster XIVa and IV, have been found to be favored by prebiotic supplementation, and negative correlations were also found with some pathogenic bacteria [3,5]. These results indicate more global changes associated with prebiotics consumption than just the effects upon bifidobacteria and lactobacilli and underline the importance of considering the total microbiota when screening compounds for their prebiotic properties. 

On the other hand, the metabolism of prebiotics leads to the generation, as main end-products, of bacterial fermentation, of short-chain fatty acids (SCFA), among which are acetate, butyrate, propionate and also branched SCFA (BSCFA: iso-valerate and iso-butyrate) and gases hydrogen, methane and carbon dioxide [6]. These compounds are well-known mediators of the microbiota-host interaction, playing an important role in host health [7,8].

To date, the most widely studied prebiotics include fructooligosaccharides (FOS) of variable chain lengths, commercial preparations often containing a mixture of molecules [9], and galactooligosaccharides (GOS), which are being often used in studies focusing on infants [10]. However, the comparative studies on the impact of different prebiotics upon the microbiota in different population groups are still scarce [11]. In this context, the assessment of the impact of prebiotics in microbiota composition and metabolism using in vitro models as a tool for screening the most effective modulatory strategies prior to accomplish expensive and complex human interventions, is valuable [12]. In vitro models, such as fecal cultures, are broadly used. Moreover, complementing such models with gas production assessment can be used for determining the fermentation profile of prebiotics by the gut microbiota of different population groups [13,14,15,16,17,18]. Among these, obese subjects constitute an interesting target, since some studies have shown that the use of prebiotics is an effective modulatory strategy in obesity [19], and animal studies provide support for a potential beneficial effect on energy homeostasis and weight loss [20]. In mice, an inverse relationship has been established between the level of bifidobacteria and some features of the metabolic alterations linked to obesity (endotoxemia, fat mass and glucose intolerance) [21]. Some of these were confirmed in human studies, such as the increase of bifidobacterial levels after prebiotic treatment, with beneficial systemic consequences for obese individuals [21,22,23,24]. However, there is still limited evidence on the in vitro fermentation profiles of different prebiotic compounds by the microbiota of obese humans [25,26,27,28] and its comparison with that of normal-weight individuals. Moreover, the characteristics of the intestinal microbiota in the extreme form of obesity (morbid obesity; MOB) (BMI ≥ 40 kg/m^2^) is still not completely known [29,30]. The variability in the response of the obese population to prebiotic and probiotic supplementation in weight loss interventions [31] and the lower microbial richness generally characterizing the microbiota of obese subjects [32] points to the gut microbiota as a target for investigation in this field.

Unfortunately, most often the in vitro screenings of prebiotic substrates have failed to consider the microbiota complexity and the potential differences on the basal microbiota composition among different human groups, with few studies selecting the best-suited compounds for defined population groups [33]. The availability of fast, easy and cheap methods, considering the influence of the basal microbiota, for assessing the fermentability and specificity of potential prebiotics would be of help in the selection of prebiotics for specific applications to human groups. In this context, it is well-known that the microbiota of obese subjects is different from that of normal-weight (NW) individuals [30,34,35], suggesting that the impact of different prebiotic compounds in these human groups may also differ, making advisable selecting the best-suited compounds for each of them. Therefore, in this study, we aimed at the evaluation of fermentative dynamics of different prebiotic substrates and the assessment of their impact on the composition and metabolic activity on the intestinal microbiota of lean and extreme obese individuals.

## 2. Results

### 2.1. Gas Production and pH Variations during Fermentation

The check of gas production in real-time allowed us to assess the in vitro fermentative dynamics of the different prebiotics. The decreases in pH and the gas formed by fecal microbiotas of NW and MOB individuals after 24 h of incubation in the presence of different carbon sources are shown in Table 1. 

The highest level of cumulative gas was reached with 1-kestose in both groups of individuals and the lowest with inulin (Table 1). Notably, in fecal cultures of MOB subjects, all prebiotics led to similar gas production (*p* > 0.05), whereas fecal cultures from NW adults showed higher heterogeneity, with significant differences in production among several substrates. The determination of kinetic parameters by the modified-Gompertz equation confirmed different dynamics of gas production between fecal cultures of MOB and NW subjects. For all prebiotics, production rates were lower in fecal cultures of MOB individuals (Table 1). In accordance with the results obtained from gas production, inulin was the substrate inducing the lower decline in pH in both population groups. Interestingly, the drops in pH did not totally mirror the increases in gas production, suggesting that differences among prebiotics are not only due to differences in their utilization yields but may also involve different microorganisms or catabolic pathways.

### 2.2. Impact of Prebiotics on Microbiota Composition

**Figure 1:**
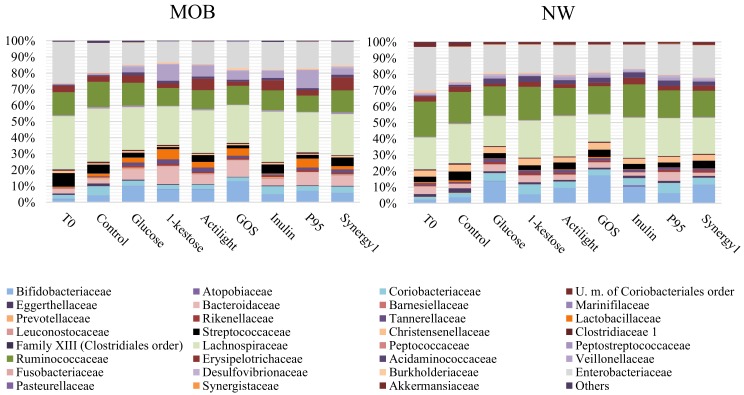
Microbial composition (relative abundance %) evaluated by 16S rRNA gene profiling at family levels in basal conditions (time 0: T0) and after 24 h of incubation in fecal cultures with several carbon sources and without an external carbon source added (Control) in morbid obesity (MOB) and normal-weight (NW) groups.

**Figure 2:**
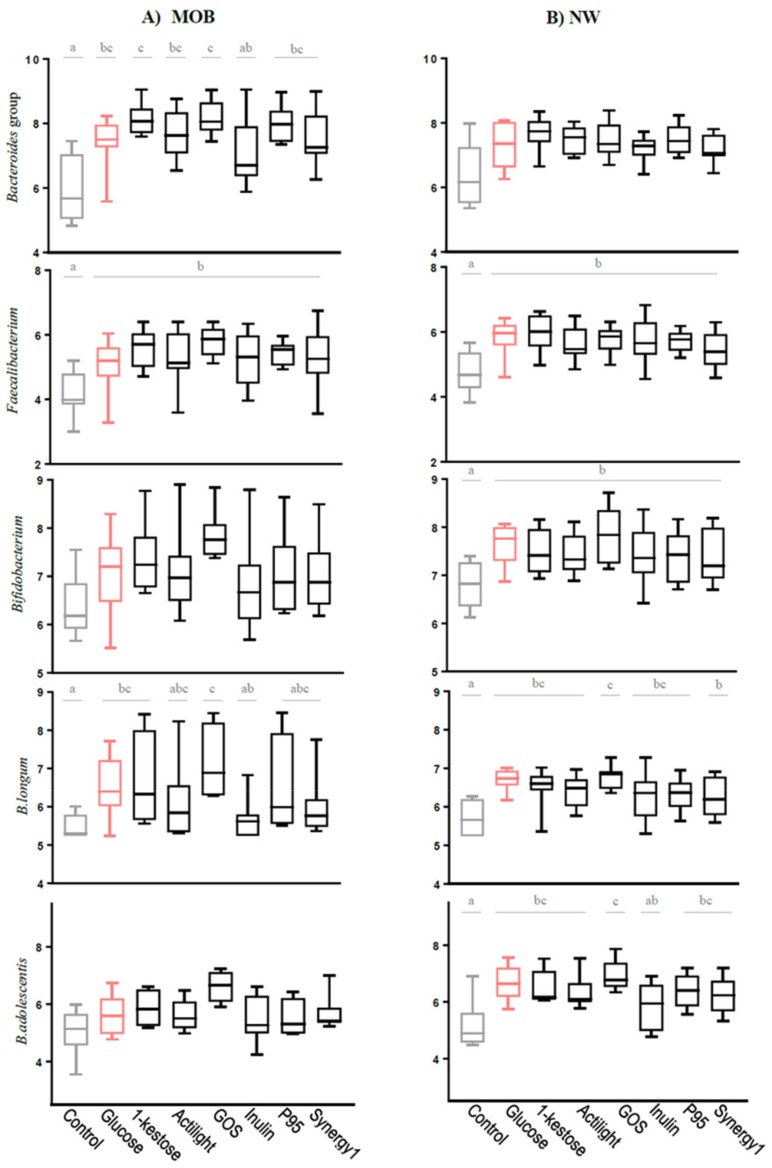
Absolute levels (Log CFU/mL) of fecal microbial groups determined by qPCR after fecal cultures of (**A**) MOB and (**B**) NW subjects. For each microbial group, the box and whiskers plot represent median, interquartile range and minimum and maximum values obtained in each human group (NW or MOB). Different letters above the boxes indicate significant differences (*p*-value < 0.05) among carbon sources for the microbial groups considered.

The microbiota composition was evaluated at the relative (16S rRNA gene profiling; Figure 1) and absolute level (quantification of representative microbial groups by qPCR; Figure 2) before (time 0) and after 24 h of incubation of fecal cultures with the carbohydrates.

The assessment of the microbial composition of fecal preparations before incubation (time 0) evidenced a high variability (Supplementary Table S1), which is an inherent feature derived from the different microbiota composition of fecal donors [36]. In spite of this, the carbon sources tested (prebiotics and glucose) displayed differential effects on the microbiota, as depending on the substrate itself and on the groups of fecal donors, MOB or NW. 

Regarding the comparison among prebiotics, in fecal cultures of MOB subjects, none of the compounds affected the overall microbiota composition, without noticing any statistically significant differences among them at phyla levels (Supplementary Table S1). At family levels, significant differences with regard to the control culture were found for some minority microbial groups. These included reduced levels of the family Eggerthellaceae, belonging to Actinobacteria phylum, and an increase of the Tannerellaceae family, belonging to Bacteroidetes, in all carbon sources. Moreover, a nonsignificant trend towards higher levels of Bacteroidetes phylum and the Bacteroidaceae family were also found (Supplementary Table S1). As with regard to qPCR data, all substrates but inulin led to a significant increase of the absolute levels of the *Bacteroides* group, as compared to the control. This effect was more pronounced with 1-kestose and GOS (8.13 ± 0.51 and 8.17 ± 0.55 Log CFU/mL, respectively) (Figure 2). An increase in the absolute levels of *Faecalibacterium* were also obtained with all substrates, whereas the genus *Bifidobacterium* was not significantly affected by any prebiotic or glucose. In spite of this, when looking at specific bifidobacterial species, the absolute levels determined by qPCR of the species *Bifidobacterium longum* were found to be increased after incubation with glucose, 1-kestose and GOS (6.52 ± 0.82, 6.66 ± 1.27 and 7.13 ± 1.03 Log CFU/mL, respectively), as compared to the control (5.47 ± 0.31 Log CFU/mL) (Figure 2). Moreover, ITS-sequencing allowed detecting a decrease of the initial higher relative abundances of *Bifidobacterium animalis* subsp. *lactis* and *Bifidobacterium crudilactis* after incubation with all prebiotics tested and with glucose (Supplementary Table S1). These results indicate that in spite of no variations found at the genus *Bifidobacterium* levels, some changes occurred in the species profiles after incubation with the prebiotic carbohydrates.

Regarding the comparison among prebiotics in fecal cultures of NW people, the 16S rRNA gene profiling evidenced very low abundances of Fusobacteria and Fusobacteriaceae in the negative control, which were practically undetectable after incubation with prebiotics (Supplementary Table S1). qPCR analyses showed that all carbon sources (prebiotics and glucose) caused a significant increase of the absolute levels of *Faecalibacterium* and *Bifidobacterium* at 24 h of incubation but did not significantly affect the population of *Bacteroides* (Figure 2). In addition, the absolute levels of the species *B. longum* increased significantly in fecal cultures of NW people with all substrates tested, and the same was true for *Bifidobacterium adolescentis,* with the exception of the prebiotic inulin (Figure 2B). Moreover, ITS-sequencing evidenced a reduction of *Bifidobacterium breve* relative abundances after incubation of NW fecal cultures with all substrates assayed (Supplementary Table S1).

Focusing on the comparison among fecal cultures of NW and MOB subjects, the absolute quantification (qPCR) of the main bacterial groups evidenced significant differences in the basal microbiota composition (time 0), for most microorganisms analyzed, between MOB and NW individuals (Supplementary Table S2). Moreover, the alpha-diversity (Chao-1 index) determined with the 16S rRNA gene profiling data demonstrated a reduced diversity (*p* < 0.05) in MOB subjects, as compared to NW (132.85 ± 43.39 *vs*. 169.19 ± 21.29, respectively). In the cultures with glucose, the differences in favor of the NW cultures for the microbial groups *Akkermansia*, *Faecalibacterium*, *B. adolescentis* and *Clostridium* cluster XIVa were maintained along incubation, giving rise to significantly higher counts of these microorganisms in NW cultures, as compared to MOB. Among prebiotics, inulin contributed to maintain differences already existing in the basal population for *Bifidobacterium catenulatum* in favor of the fecal cultures of NW individuals. The genus *Akkermansia* was significantly higher at 24 h of incubation in the cultures of NW individuals with respect to MOB in all conditions, these differences being not evident in cultures of the basal microbiota with no carbohydrates added (negative control). However, the most noticeable effect among prebiotics was that promoted by GOS on the *Bacteroides* group in MOB. Interestingly, ITS analysis showed a clear differential pattern of abundances of several bifidobacterial species between fecal cultures of NW and MOB subjects after incubation with different carbon sources (Supplementary Figure S1). The comparison between fecal samples of both human population groups in basal conditions (time 0, before incubation) reflected higher abundances of *Bifidobacterium mongoliense* and *B.crudilactis* in NW individuals (2.78 ± 4.13 % and 14.98 ± 17.04 %, respectively) than in MOB subjects (0.16 ± 0.16 % and 1.96 ± 1.46 %, respectively) and a lower species richness (number of species of bifidobacteria detected by ITS-sequencing) in the NW fecal cultures at time 0 and after 24 h incubation in all conditions assayed (Supplementary Table S3). Moreover, the qPCR quantification of bifidobacterial species confirmed higher (*p* < 0.05) levels of *B. longum* (NW: 5.85 ± 0.48; MOB: 5.60 ± 0.32) and *B. catenulatum* (NW: 6.80 ± 0.70; MOB: 5.34 ± 1.10) in NW subjects. All these results point to substantial differences at the species level in the microbiota of NW and MOB subjects that are conditioning differences among fecal cultures from groups NW and MOB subjects after the incubation with prebiotics. 

### 2.3. Production of Short-chain Fatty Acids

**Figure 3:**
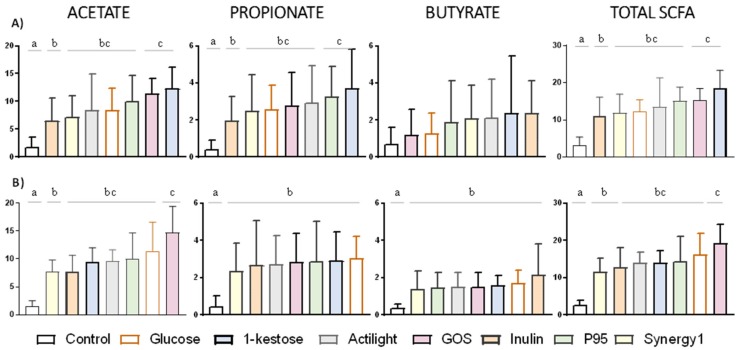
Increments in ascending order, with respect to time 0, in the concentration (mM) of the major short-chain fatty acids (acetic, propionic and butyric) after 24 h of incubation with different carbon sources in fecal cultures from MOB (**A**) and NW (**B**) groups. Differences are shown for each short-chain fatty acid (SCFA); columns that do not share the same letter are significantly different (*p* < 0.05).

In a similar way as for microbiota composition, differences on the levels of SCFA were determined in fecal cultures depending on the prebiotic tested and the population group considered (Figure 3). Focusing on the comparison among prebiotics, GOS and 1-kestose were the substrates promoting the highest increase of total SCFA at 24 h of incubation in NW and MOB fecal cultures, respectively. 1-kestose gave rise to the highest increase of acetate and propionate among prebiotics tested in fecal cultures of MOB subjects, whereas GOS was the main promoter of acetate production in cultures from NW individuals. Inulin and Synergy1 were the prebiotics with a lower impact on the production of acetate and propionate in fecal cultures of both MOB and NW people. All prebiotics enhanced butyrate production in fecal cultures of NW individuals, with no clear differences among the different compounds (Figure 3B). In contrast, not statistically significant increases of butyrate were evidenced in fecal cultures of MOB subjects (Figure 3A), which could be due to the high variability in the production of this compound by the fecal cultures analyzed. In spite of that indicated above, no significant differences were obtained in the increments of acetic, propionic, butyric, BSCFA and total SCFA between fecal cultures of MOB and NW individuals at 24 h of incubation (Mann Whitney U test, *p*-value > 0.05).

### 2.4. Interaction of the Isolated Microbiotas and Supernatants from the Fecal Cultures with HT29 Cells

**Figure 4:**
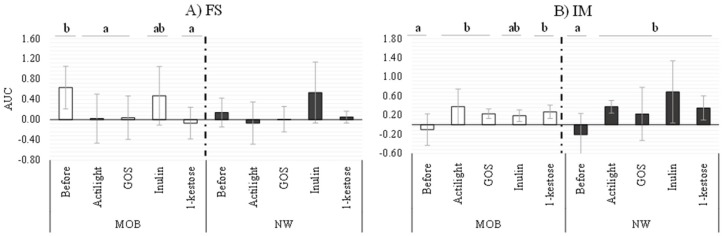
Real-time monitoring the interaction between (**A**) fecal supernatants and (**B**) isolated microbiota obtained before and after incubation with prebiotics and HT29 intestinal epithelial cells. Values (media ± SD) correspond to the Area Under the Curve (AUC) resulting from monitoring the cell index (CI) during 10 h. Significant differences (*p*-value < 0.05) represent the comparison of results before and after prebiotics addition in each condition.

Functional differences of fecal supernatants (FS) and isolated microbiotas (IM) collected before and after incubation with representative prebiotics (1-kestose, Actilight, inulin and GOS) were evaluated through an in vitro model using the HT29 intestinal cell line (Figure 4). Regarding FS, a significant decrease of the Area Under the Curve (AUC) values was evidenced after incubation of the HT29 cell line with samples from fecal MOB cultures added with prebiotics (AUC values ranging between −0.07 and 0.04), with respect to the value before the addition of substrates (0.63 ± 0.42). The only exception to this was inulin, for which no significant variations were obtained (Figure 4A). It is interesting to note that AUC determined with FS from cultures of MOB subjects added with prebiotics resemble those obtained with FS of NW subjects. Notably, in these last samples, the AUC before incubation with HT29 were lower than in MOB cultures. These data suggest that the functionality of the FS from MOB subjects could be restored (becoming similar to that of NW subjects) after culturing with some of the prebiotics studied (1-kestose, Actilight and GOS). In co-cultures of HT29 with IM from fecal cultures of MOB and NW groups, all prebiotics promoted significant increases of AUC values, with the exception of inulin in both population cohorts.

## 3. Discussion

The definitive way to prove the impact of prebiotics on gut microbiota and health is through human intervention studies. However, these studies are expensive and time-consuming, and it is advisable to perform an initial screening of the different candidate substrates by using affordable in vitro models that could help to predict in vivo functionality [12]. In a previous work, we described an in vitro model that allowed us to predict the functionality of IM and FS of different human populations groups [37]. Its application in the present work for assessing the impact of several prebiotics in NW and MOB microbiota highlighted potential modulatory benefits on the gut microbiota of some FOS and GOS. Particularly, real-time monitoring the interaction with the HT29 intestinal cell line of FS from fecal cultures of MOB subjects added with different prebiotics evidenced a response that approaches values obtained of FS from NW cultures in the same conditions. It suggests a possible restoration of the unbalanced functionality of the microbiota by some of the prebiotic substrates tested. Nevertheless, inulin preserved a behavior more similar to the initial situation in NW and MOB groups, probably due to the fact that inulin was the prebiotic with less marked effects on microbiota composition and activity, which is consistent with previous reports by other authors [14,16].

Another functional approach tested in this study was the application of a gas monitoring profiling system during fecal cultures of NW and MOB microbiotas with different prebiotics. Although this system has been recently applied to human fecal cultures [18,38], this is the first report on their use to prebiotics evaluation. Differences in gas production ability among prebiotic substrates can be partly explained by possible differences in the fermentability among compounds but also by differential effects of these substrates on the intestinal microbiota. In fact, prebiotics may differ in their ability to modulate the growth and activity of those microorganisms with limited gas production and/or to up-regulating microbial gas-consuming reactions (methanogenesis, sulfate reduction and acetogenesis) [39]. In addition to the potential direct impact on gas-producing bacteria, prebiotics are also known to affect other microorganisms of the intestinal microbiota, such as bifidobacteria, not releasing gas but producing acetate and lactate; these compounds could be involved in cross-feeding mechanisms with gas-producing microorganisms such as *Clostridium* spp. or sulphate-reducing bacteria [40]. Therefore, the final gas formed will depend, not only on the chemical and physical structure of the prebiotic, but on several other factors related with the composition and metabolic activity of the intestinal microbiota. In this context, different fermentation dynamics of fecal cultures from MOB and NW people were demonstrated in this study. A lower rate of gas production was appreciated with the MOB microbiota in all tested carbon sources. This feature suggests a metabolically less active microbiota.

In the context of obesity, an inverse association between body mass index and H_2_ and CH_4_ gas detection in breath tests has been reported [41,42], which is in good agreement with the lower cumulative gas in MOB cultures obtained herein. Although emphasis has been given to the potential inflammatory or carcinogenic properties of colonic gases, emerging evidence suggests that these gases might have a beneficial effect in colonic health [43]. One of the main gases produced by anaerobic fermentation is H_2_ [44], and an imbalance in its metabolism (H_2_-producing and H_2_-consuming bacteria) might facilitate inflammation [43]. It is intriguing to consider whether the promotion of gas production in MOB subjects could improve antioxidant and antiapoptotic status than contributing to decrease inflammation [45].

The prebiotics used in the present work have previously proven efficacy for modulating the microbiota of the general or specific population groups, both in in vivo and in vitro models [21]. In our case, the most pronounced effects in NW and MOB fecal cultures were obtained with 1-kestose and GOS. In this way, 1-kestose has been found to be metabolized by different intestinal microorganisms [46,47]. Moreover, a recent study reported that the administration to healthy volunteers of 1-kestose (5 g/day) during eight weeks promoted an increase of the intestinal populations of *Faecalibacterium prausnitzii* and *Bifidobacterium* spp. [48]. The potential beneficial changes promoted by GOS and FOS on the intestinal microbiota found in the present work are in good agreement with the widely reported effects of these substrates on the microbiota composition of the general population, assessed in clinical trials and used in vitro models [21,49].

It is important to emphasize that in the present work we have focused on morbid obese subjects, which could make the comparison with other studies difficult since, in the literature available, obese individuals are not often subcategorized. Moreover, the obesity-associated microbiota shifts are still not completely known, and several confounding factors often make this task difficult [32,50]. In this regard, the modulation of the microbiota of obese subjects by prebiotics has produced contradictory results on the genus *Bacteroides*, with increases reported after the administration of α-glucooligosaccharide and arabinogalactan [25,26] and decreases with FOS [23]. Discrepancies on experimental results are likely due to a differential effect depending on the type of prebiotics, the experimental design and the analytical techniques used to determine the composition of the intestinal microbiota by different authors. It is also necessary to point out that in the present work all prebiotics tested were able to up-modulate the levels of the genus *Faecalibacterium*, a microorganism with well-known anti-inflammatory properties [51] and, therefore, interesting in the context of obesity, which is generally accompanied by a low-grade inflammation [52,53]. A bifidogenic effect was observed only for the species *B. longum* after the addition of the prebiotics 1-kestose and GOS to fecal cultures. These observations and the inverse association of this species with serum lipopolysaccharides and endotoxemia [22,54] suggests that some of these prebiotics, specially 1-kestose and GOS, could be good candidates to modulate the microbiota in the context of obesity. 

A broad prebiotic enhancement of absolute levels of the genus *Bifidobacterium* was seen in fecal cultures of the NW group, in contrast to the absence of effect in cultures from MOB subjects. In order to expand the study of the prebiotic impact on bifidobacteria, we performed an ITS-region profiling. To our knowledge, this is the first study going in detail on bifidobacteria species variation in NW and MOB fecal cultures. Even though, regarding the influence of prebiotics, the ITS-profiling only stood out as a decrease after the supplementation with carbon sources of *B. animalis* subsp. *lactis* and *B. crudilactis* in NW cultures and of *B. breve* in cultures of MOB subjects, a clear distinction between MOB and NW microbiotas was still evident. Firstly, we noticed a greater richness of bifidobacteria species in MOB microbiota. Additionally, differentially higher levels of *B. monogoliense* and *B. crudilactis* were present in the microbiota of the NW group, which may be explained by a higher consumption of dairy fermented foods [55,56].

The production of SCFA in fecal cultures is in good agreement with variations in pH, gas production and impact caused on the microbiota composition by the different substrates. Thus, differences in the production of propionate between fecal cultures of MOB and NW promoted by 1-kestose could be directly related with its higher capacity (together with GOS) to differentially promote the increase of *Bacteroides* (the main propionate producer in the human colon and a producer of acetate) in fecal cultures of MOB individuals. Interestingly the intestinal microbiota, mainly that from MOB subjects, showed a better ability to produce SCFA with some of the prebiotics tested than from glucose. This could be related with the enrichment of the microbiome in some metabolic pathways involved in the initial steps of breaking down indigestible dietary polysaccharides, including pathways for starch/sucrose metabolism, galactose metabolism and butanoate metabolism, previously reported in the obese population [57]. 

It is also worth mentioning that the basal differences in the metabolic activity and microbiota composition, added to the specific effects of prebiotics found in the present work depending on the donor population, highlights the importance of selecting the best-suited compounds for the desired target population and the potential limitations of extrapolating conclusions from one population group to another.

To summarize, the present study provides evidence about the in vitro fermentation profiles of different prebiotics by microbiotas from NW and MOB individuals. In our study, we did not perform total metagenome analyses, but instead, we performed a microbiota characterization by 16S rRNA gene profiling and complemented it with two functional tests, gas production and interaction with an intestinal line, allowing the assessment of both microbiota compositions and some functional properties of these microbiotas. By monitoring gas production along fermentation, we found a higher capacity of gas production by fecal cultures of NW subjects than from MOB individuals. 1-kestose was the fructan showing the highest gas accumulation and largest microbiota modulation activity in MOB subjects, together with GOS, underlining the utilization of this compound by the intestinal microbiota of these individuals. The fecal cultures incubated with some of the prebiotics tested also showed differences at the functional level when assessed upon epithelial cell lines. Even though the in vitro models present inherent limitations and a difficult interpretation with respect to physiological conditions, the application of in vitro models to the analysis of microbiota composition and functionality could allow the selection of the most suitable prebiotics for different populations prior to their assessment in human intervention studies. Moreover, our results underline the interest of further exploring the prebiotic role of 1-kestose due to their modulatory capacity of the microbiota composition and activity in MOB subjects.

## 4. Materials and Methods 

### 4.1. Prebiotics and Carbon Sources 

Two types of prebiotics, based on their monosaccharide’s composition (fructose or galactose), were evaluated. Among FOS, the trisaccharide 1-kestose (>99%; β Food Science Co. Ltd., Chita, Japan); Actilight^®^ (DP = 3–5, enzymatically produced, 95% purity; Beghin Meiji, Lila, France); P95 (DP = 2–8, obtained by hydrolysis, 95% purity; Beneo-Orafti, Oreye, Belgium); Synergy1 (FOS plus inulin in proportion 1:1, 92% purity; Beneo-Orafti, Oreye, Belgium) and the long-chain fructan–inulin (DP>36; Sigma-Aldrich, Madrid, Spain) extracted from dahlia tubers were included in the study. A GOS from the brand Bimuno Daily (Clasado, Shinfield, England) with 79.70% (*w*/*w*) of purity was also evaluated. Glucose (Fluka Analytical, Madrid, Spain) was also used as a nonprebiotic universal carbon source. Sterilization of all substrates was carried out by filtration through a pore size of 0.45 μm, except for the inulin, which was autoclaved. 

### 4.2. Volunteers and Fecal Sample Collection

Fecal samples were obtained from nine healthy NW adults (77.78% women; BMI <25 kg/m^2^) and nine MOB volunteers (75% women; BMI ≥40 kg/m^2^) recruited at the Digestive and Endocrinology Services, respectively, of Asturias Central University Hospital (HUCA, Asturias, Spain). The mean age of the volunteers was 38 ± 9 and 45 ± 10 for NW and MOB subjects, respectively. All participants have followed an unrestricted diet and have not taken antibiotics during the previous six months. The study was approved by the Regional Ethical Committee of Asturias Public Health Service (Ref. Nº 120/13, 20 November 2013), and an informed written consent was obtained from each volunteer. Samples were collected and immediately introduced into anaerobic jars (Anaerocult A System; Merck, Darmstadt, Germany) for transportation to the laboratory within 1 h after collection. A 1/10 (*w*/*v*) dilution was made in prereduced PBS solution and homogenized in a Lab-Blender 400 stomacher (Seward Medical, London, UK) for 5 min.

### 4.3. Fecal Batch Culture Fermentation 

Independent batch fermentations were performed at pH-uncontrolled in a carbohydrate-free basal medium (CFBM) [58], with feces from different human donors and different carbohydrates added. Briefly, CFBM was prepared and reduced overnight in an anaerobic chamber MG500 (Don Whitley Scientific, West Yorkshire, UK) one day before the sample processing. On the day of the assay, fresh fecal samples, collected and homogenized as stated above, were added (10% *v*/*v*) to the reduced CFBM and then were distributed into 100 mL bottles of the ANKOMRF system (ANKOM Technology, USA). An overnight incubation in anaerobic conditions was performed at 37 °C prior to the addition of carbon sources in order to allow microbiota to stabilize in the culture medium. 

A set of independent fermentations were performed with feces from each donor, using as carbon sources either inulin-type fructans (1-kestose, Actilight, P95, Synergy1 and inulin), GOS or glucose (nonprebiotic positive control) at a final concentration of 0.3% (*v*/*v*). A bottle with no carbon source added was used as a control. Fermentations were carried out under anaerobic conditions at 37 °C during 24 h. The pH of cultures was determined with a pHmeter (SensION + PH3, Hach; Barcelona, Spain) and was considered as an indicator of the progression of fermentation. Samples (1 mL) were taken in duplicate before incubation (time 0) and considered as basal conditions and at 24 h of incubation. Samples were centrifuged at full speed for 10 min, and supernatants and pellets were stored separately at −20 °C until their use for microbiota and metabolite analyses.

### 4.4. Gas Monitorization

The cumulative gas produced along the different fermentations was monitored in real-time by using the ANKOM RF system. The system provides increases in pressure (psi), which can be converted to mL of gas produced using the ideal gas equation.

V = Vj · Ppsi · 0.068004084(1)

where V = gas volume at 39 °C in mL, Vj = headspace of digestion jar (glass bottle) in mL and Ppsi = cumulative pressure recorded by Gas Monitor System software.

The data of gas production were fitted to a modified-Gompertz equation, a model frequently used to fit data of bacterial, plant growth, tumor proliferation and gas production [59], by using the formula:

(2) $$y=A\times \exp\left\{-\exp\left[\frac{µ\times e}{A}\left(\lambda -t\right)+1\right]\right\}$$

in which variable “A” represents the upper asymptote (mL), “µ” is the rate of gas production (mL/h) and “λ” is the time lag before the exponential phase (h).

### 4.5. Microbiota Composition and SCFA Quantification

DNA was extracted from the pellets harvested using the QIAamp DNA Stool Mini kit (Qiagen GmbH; Hilden, Germany), as previously described [60], and the isolated DNA was stored at −20 °C until use for qPCR analyses and 16S ribosomal and intergenic ribosomal transcriber spaces (ITS).

#### 4.5.1. qPCR Analyses

Absolute levels of some relevant intestinal microbial groups (*Bacteroides–Prevotella–Porphyromonas* group, *Lactobacillus* group, *Akkermansia*, *Clostridium* cluster XIVa, *Bifidobacterium* and *Faecalibacterium* genus), as well as total bacteria, were determined at 0 and 24 h of fermentation by qPCR using previously described primers and conditions [31,61]. Variations in the levels of the species *B. longum*, *B. catenulatum* and *B. adolescentis* were assessed as described elsewhere [22].

#### 4.5.2. 16S rRNA Gene Based Microbiota Profiling

Purified DNA was used as a template for amplification of partial 16S rRNA gene sequences by PCR using the primers and conditions described by Milani and coworkers [62]. The obtained amplicons were then sequenced by using the MiSeq (Illumina) platform at GenProbio srl (Italy). The individual reads obtained were filtered, trimmed and processed [63]. 16S rRNA operational taxonomic units were defined at ≥ 97% sequence homology using the UCLUST tool developed by Edgar [64]. All reads were classified to the lowest possible taxonomic rank using QIIME and a reference dataset from the SILVA database [65]. 

#### 4.5.3. ITS Region-Based Profiling of Bifidobacterial Microbiota

To gain further insight into the fecal bifidobacterial populations present in the samples and how the different prebiotics affected them, the 16S–23S internal transcriber spaces of the ribosomal DNA (ITS region) was amplified by PCR using the primer pair Probio_bif_uni/Probio_bif_rev. and further sequenced as indicated in the previous section. An improved bifidobacterial ITS database, containing all publicly available bifidobacterial genomes and a custom bioinformatics script [66], were used. Relative abundance of bifidobacterial composition filtered by a minimum presence (≥1%) of each species in all databases were represented by a heatmap following instructions described elsewhere [67], centered and scaled by the “scale” function in RStudio version 1.2.5001.

#### 4.5.4. SCFA Analyses

The analysis of SCFA was performed by gas chromatography (GC) in the fecal culture supernatants in order to determine the molar concentrations of three main compounds: acetate, propionate and butyrate. The remaining BSFA, namely isobutyrate and isovalerate, were also quantified and summed up for further analysis. Briefly, culture supernatants collected during the fecal fermentation (0.250 mL) were mixed with 0.3 mL methanol, 0.05 mL internal standard solution (2-ethylbutyric 1.05 mg/ mL) and 0.05 mL of 20% formic acid. This mixture was centrifuged, and the supernatant was used for quantification of SCFA by GC, as described previously [37]. Samples were analyzed in triplicate. Increments in molar concentrations of the main SCFA and BSCFA with respect to the time 0 were calculated for each fermentation batch with the different carbon sources.

### 4.6. Monitoring the Interaction of Isolated Microbiotas (IM) and Fecal Supernatants (FS) with HT29 Cells

Briefly, the behavior of HT29 cells monolayers in a confluent state was assessed upon exposure to IM and FS collected after incubation of fecal samples with different carbon sources by using a real-time cell analyzer (RTCA-DP) xCelligence apparatus (ACEA Bioscience Inc., San Diego, CA, USA). The culture conditions and the maintenance of the intestinal epithelial cell line HT29 (ECACC 91072201) are detailed in a previous work where the functional model was developed [37]. IM were purified from 10-fold concentrated fecal cultures by using a density gradient method previously described [68]. Purified microbiotas were inactivated by UV light exposure (15 W; Selecta, Barcelona, Spain) and adjusted to 1 × 10^8^ bacteria/mL using a Neubauer-improved camera (Blau Brand, Germany).

For the functional assessment of IM and FS, HT29 monolayers in a confluent state were coincubated with 6.5 × 10^7^ bacteria/mL of UV-inactivated purified microbiotas in McCoy´s medium (MM) (bacteria to cell ratio 10:1). In the case of fecal supernatants, the behavior of HT29 cells monolayers was assessed with filtered fecal supernatants (pH adjusted to 7.55 ± 0.05) and diluted 40% with MM. Additionally, a negative control consisting of MM without bacteria or fecal supernatants was included in each experiment. Each sample was tested in duplicate using two independent E-plates. The monitoring was followed for every 10 min under standard incubation conditions. CI values recorded were normalized by the time of the sample addition and by the control sample, as previously described [69]. For statistical comparison purposes, the “Area Under the Curve” (AUC), representing the CI values along 10 h of incubation for each sample, was calculated as explained in [37].

### 4.7. Statistics Analyses

Unless otherwise specified, all experimental data are reported as mean ± standard deviation. Statistical analysis of results was performed using the software SPSS v.25 (SPSS Inc., Chicago, USA). Data were compared for the effect caused on the parameters analyzed by the addition of different carbon sources in fecal cultures from each population cohort (NW and MOB) at the end of fermentation (24 h). For variables with a normal distribution (Shapiro-Wilk test) and homoscedasticity (Levene test), one-way ANOVA followed by post hoc LSD comparison were conducted (Supplementary Table S4). In the remaining cases (variables showing non-normal distribution), a Kruskal-Wallis test followed by a post hoc Dunn’s test of pairwise comparisons were applied when necessary (Supplementary Table S4). A significant *p*-value of 0.05 was used for the interpretation of results. For two-group comparisons between MOB and NW (at time 0 and after incubation with all conditions), a two-tailed Student’s *t*-test or Mann-Whitney *U* test was conducted for the evaluation of data by parametric or nonparametric contrast, respectively. 

## Figures and Tables

**Table 1:** Cumulative gas produced (mL) and decreases of pH values (Δ pH) after 24 h of incubation in fecal cultures with normal-weight (NW) and morbid obesity (MOB) microbiota. Kinetic parameters were determined using the modified-Gompertz equation, in which “A” represents the upper asymptote (mL) and “µ” is the rate of gas production (mL/h). The values not sharing the same superscript (a, b, c or d) indicate significant differences (*p*-value < 0.05) among carbon sources for each population group (NW or MOB).

Group	Condition	Δ pH	Cumulative Gas	A	µ	R^2^
MOB	Control	0.10 ^a^ ± 0.06	5.10 ^a^ ± 0.65	5.111	0.39	0.979
	Glucose	−1.22 ^b^ ± 0.43	18.44 ^b^ ± 7.08	19.306	1.205	0.999
	1-kestose	−1.34 ^b^ ± 0.20	21.05 ^b^ ± 5.09	22.299	1.267	0.997
	Actilight	−1.37 ^b^ ± 0.18	19.80 ^b^ ± 5.40	20.486	1.163	0.998
	GOS	−1.37 ^b^ ± 0.20	19.24 ^b^ ± 4.89	20.394	1.293	0.998
	Inulin	−0.86 ^a^ ± 0.17	17.68 ^b^ ± 8.54	21.915	0.822	0.997
	P95	−1.25 ^b^ ± 0.21	19.82 ^b^ ± 5.82	21.688	1.294	0.997
	Synergy1	−1.18 ^b^ ± 0.10	18.48 ^b^ ± 8.32	18.68	1.189	0.997
NW	Control	0.07 ^a^ ± 0.10	5.52 ^a^ ± 1.84	5.421	0.364	0.979
	Glucose	−1.16 ^c^ ± 0.31	25.62 ^c,d^ ± 6.38	27.399	1.589	0.999
	1-kestose	−1.28 ^c^ ± 0.16	26.57 ^d^ ± 5.87	27.52	1.861	0.999
	Actilight	−1.25 ^c^ ± 0.20	19.73 ^b,c^ ± 6.36	20.635	1.384	0.998
	GOS	−1.28 ^c^ ± 0.25	22.20 ^b,c,d^ ± 5.63	22.641	1.761	0.998
	Inulin	−0.77 ^a,b^ ± 0.23	19.10 ^b^ ± 6.51	21.662	0.962	0.997
	P95	−1.24 ^c^ ± 0.16	23.67 ^b,c,d^ ± 5.76	23.952	1.721	0.998
	Synergy1	−1.08 ^b,c^ ± 0.13	25.19 ^b,c,d^ ± 6.25	26.454	1.456	0.997

## Supplementary Materials

Supplementary materials can be found at https://www.mdpi.com/1422-0067/21/3/906/s1.

## Abbreviations

AUC	Area Under the Curve
CI	Cell Index
FS	Fecal Supernatant
GC	Gas Chromatography
IM	Isolated Microbiota
ITS	16S-23S internal transcriber spaces
LSD	Least Significant Difference post-hoc
MM	McCoy´s medium
MOB	Morbid Obesity
NW	Normal-Weight Adults
qPCR	Real-time polymerase chain reaction
rRNA	Ribosomal ribonucleic acid
RTCA	Real-time Cell Analyzer
