# Supplementary material for: In Vitro Evaluation of Different Prebiotics on the Modulation of Gut Microbiota Composition and Function in Morbid Obese and Normal-Weight Subjects
Source: Int J Mol Sci. 2020 Jan 30;21(3):906. doi: 10.3390/ijms21030906 (PMC7038051; doi:10.3390/ijms21030906)
Supplement: Supplementary file 1 [file ijms-21-00906-s001.pdf]

Figure S1. Heatmap representing *Bifidobacterium* species obtained by ITS-region profiling whose relative abundances were ≥1% in fecal cultures of NW and MOB subjects added with different carbon sources and without any carbohydrate added (control) after 24 h of incubation. Tiles are colored based on the scaled abundance of each species, red and grey colors indicating high or low abundances, respectively. Each row represents the media of bifidobacterial composition in each population group after each fecal culture experiment. The data were indicated by the first vertical colored bars at the left representing NW (blue) or MOB (red) subjects, and the second vertical colored bars at the left representing different carbohydrates.

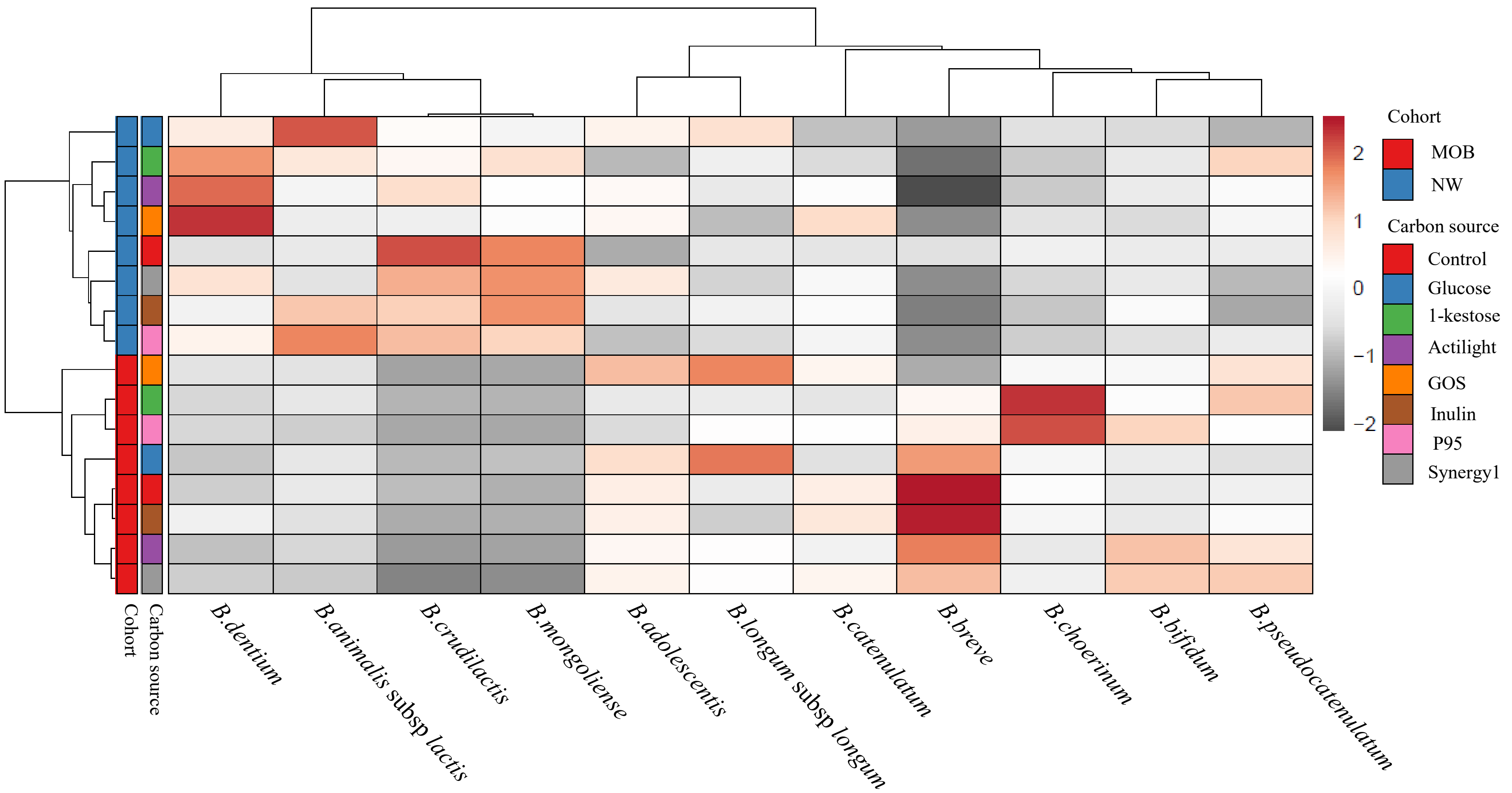

Table S1. Relative abundances determined by 16S rRNA gen profiling at phylum and family level and ITS-region profilng of *Bifidobacterium* species after 24 h of incubation with different carbon sources and without any carbon source added (control) where p-value <0.1. Different letters indicate significant differences (p <0.05) among carbon sources and control after 24 hours of fermentation for the microorganisms considered. Information of relative levels at basal conditions (T0) for all groups is provieded.

			T0	T24							
Group	Taxonomic rank	p-value		Control	Glucose	1-kestose	Actilight	GOS	Inulin	P95	Synergy1
MOB	Phylum										
	Bacteroidetes	0.056	2.34 [1.44 - 4.56]	3.51 [1.63 - 6.24]	12.06 [5.52 - 15.29]	13.46 [6.89 - 24.72]	7.32 [3.77 - 15.47]	12.10 [5.31 - 20.90]	8.23 [2.23 - 10.63]	13.74 [4.88 - 14.79]	6.84 [2.84 - 18.05]
	Family										
	Eggerthellaceae	0.013	0.57 [0.26 - 1.10]	1.02 [0.73 - 2.50][b]	0.44 [0.28 - 0.69][a]	0.36 [0.21 - 0.53][a]	0.23 [0.13 - 0.41][a]	0.33 [0.17 - 0.47][a]	0.30 [0.16 - 0.79][a]	0.35 [0.28 - 0.45][a]	0.48 [0.27 - 0.55][a]
	Bacteroidaceae	0.081	1.48 [0.76 - 2.31]	1.79 [0.84 - 4.91]	5.53 [2.79 - 10.31]	10.89 [2.96 - 16.23]	5.25 [2.03 - 9.98]	8.78 [2.29 - 17.98]	4.85 [1.05 - 6.68]	10.85 [1.68 - 11.03]	4.68 [1.31 - 11.58]
	Prevotellaceae	0.076	0.20 [0.05 - 0.38]	0.52 [0.17 - 1.01]	0.30 [0.12 - 0.72]	0.05 [0.02 - 0.18]	0.16 [0.04 - 0.71]	0.20 [0.12 - 0.39]	0.55 [0.25 - 1.41]	0.19 [0.02 - 0.37]	0.26 [0.14 - 0.69]
	Tannerellaceae	0.012	0.14 [0.07 - 0.25]	0.16 [0.09 - 0.50][a]	1.34 [0.82 - 2.80][b]	1.37 [0.82 - 4.50][b]	1.29 [0.67 - 3.11][b]	1.02 [0.79 - 2.63][b]	0.57 [0.22 - 1.51][b]	0.98 [0.55 - 3.16][b]	1.07 [0.45 - 3.07][b]
	Bifidobacteria ITS										
	B. animalis subsp *lactis*	0.020	1.07 [0.45 - 1.39]	0.59 [0.47 - 1.23][c]	0.15 [0.02 - 0.57][b]	0.31 [0.12 - 0.73][bc]	0.24 [0.09 - 0.63][abc]	0.05 [0.00 - 0.10][a]	0.57 [0.16 - 1.09][bc]	0.40 [0.11 - 0.65][abc]	0.37 [0.20 - 0.43][bc]
	B. crudilactis	0.022	2.12 [0.82 - 3.03]	1.62 [0.93 - 2.41][c]	0.29 [0.12 - 1.52][ab]	0.50 [0.20 - 2.14][abc]	0.49 [0.32 - 1.04][ab]	0.09 [0.06 - 0.12][a]	1.15 [0.41 - 2.61][bc]	0.45 [0.25 - 1.54][abc]	0.49 [0.30 - 1.27][bc]
NW	Phylum										
	Fusobacteria	0.04	0.01 [0.00 - 0.04]	0.02 [0.00 - 0.05][b]	0.01 [0.00 - 0.01][ab]	0.00 [0.00 - 0.00][a]	0.00 [0.00 - 0.01][a]	0.00 [0.00 - 0.01][ab]	0.00 [0.00 - 0.01][a]	0.00 [0.00 - 0.00][a]	0.00 [0.00 - 0.00][a]
	Family										
	Fusobacteriaceae	0.03	0.01 [0.00 - 0.04]	0.02 [0.00 - 0.05][b]	0.01 [0.00 - 0.01][ab]	0.00 [0.00 - 0.00][a]	0.00 [0.00 - 0.00][a]	0.00 [0.00 - 0.01][ab]	0.00 [0.00 - 0.01][a]	0.00 [0.00 - 0.00][a]	0.00 [0.00 - 0.00][a]
	Others	0.02	0.19 [0.16 - 0.41]	0.33 [0.23 - 0.78][b]	0.13 [0.08 - 0.24][a]	0.12 [0.05 - 0.24][a]	0.14 [0.10 - 0.26][a]	0.18 [0.11 - 0.27][ab]	0.13 [0.07 - 0.24][a]	0.10 [0.05 - 0.13][a]	0.10 [0.06 - 0.20][a]
	Bifidobacteria ITS										
	B. breve	0.01	0.29 [0.03 - 0.59]	0.51 [0.33 - 1.10][b]	0.00 [0.00 - 0.27][a]	0.10 [0.00 - 0.27][a]	0.11 [0.00 - 0.29][a]	0.00 [0.00 - 0.12][a]	0.00 [0.00 - 0.09][a]	0.19 [0.02 - 0.27][ab]	0.00 [0.00 - 0.27][a]

Table S2. Absolute levels (mean ± SD) (Log UFC/mL) of fecal microbial groups and some *Bifidobacterium* species determined by qPCR at basal conditions (T0) and after 24 hours of incubation with different carbon sources. The comparison of both population groups for each carbon source is shown with "*" (p<0.05).

Condition	Group	*Lactobacillus-group*	*Akkermansia*	*Bacteroides* group	*Faecalibacterium*	*Bifidobacterium*	*B.longum*	*B.catenulatum*	*B.adolescentis*	Enterobacteria	*Clostridium* cluster XIVa	Total
T0	MOB	**4.56 ± 0.96***	**3.18 ± 0.39***	**6.71 ± 0.88***	**4.79 ± 0.81***	**6.37 ± 0.45***	**5.60 ± 0.32***	**5.34 ± 1.10***	4.80 ± 0.67	**7.41 ± 1.27***	6.77 ± 0.62	**8.36 ± 0.58***
	NW	**3.63 ± 0.83**	**4.66 ± 1.39**	**7.04 ± 0.92**	**5.37 ± 0.84**	**6.96 ± 0.48**	**5.85 ± 0.48**	**6.80 ± 0.70**	5.15 ± 0.90	**7.90 ± 0.98**	6.62 ± 0.70	**8.66 ± 0.54**
Control	MOB	4.20 ± 1.32	3.17 ± 0.41	5.99 ± 1.01	4.18 ± 0.67	6.40 ± 0.61	5.47 ± 0.31	5.47 ± 1.24	5.05 ± 0.81	6.80 ± 1.32	5.80 ± 0.75	7.87 ± 0.61
	NW	3.82 ± 1.01	4.39 ± 1.28	6.39 ± 0.96	4.75 ± 0.63	6.78 ± 0.47	5.70 ± 0.45	6.97 ± 0.56	5.16 ± 0.90	7.84 ± 0.92	6.71 ± 0.54	8.15 ± 0.65
Glucose	MOB	4.67 ± 1.45	**3.23 ± 0.45***	7.42 ± 0.77	**5.04 ± 0.80***	7.06 ± 0.83	6.52 ± 0.82	5.67 ± 1.34	**5.69 ± 0.70***	7.16 ± 1.35	**5.41 ± 1.45***	8.26 ± 0.77
	NW	3.88 ± 1.00	**4.53 ± 1.41**	7.36 ± 0.72	**5.83 ± 0.56**	7.65 ± 0.43	6.70 ± 0.27	7.40 ± 1.01	**6.67 ± 0.63**	7.84 ± 0.94	**6.85 ± 0.42**	8.72 ± 0.39
1-kestose	MOB	5.20 ± 1.62	**3.00 ± 0.00***	8.13 ± 0.51	5.59 ± 0.60	7.37 ± 0.76	6.66 ± 1.27	6.40 ± 1.42	5.86 ± 0.67	7.79 ± 0.98	6.43 ± 1.19	8.91 ± 0.40
	NW	3.93 ± 1.16	**4.82 ± 1.31**	7.67 ± 0.52	5.98 ± 0.56	7.48 ± 0.46	6.49 ± 0.53	7.43 ± 0.88	6.50 ± 0.62	7.96 ± 1.00	6.90 ± 0.34	8.90 ± 0.33
Actilight	MOB	4.78 ± 1.47	**3.15 ± 0.28***	7.67 ± 0.75	5.26 ± 0.84	7.09 ± 0.82	6.14 ± 1.02	6.37 ± 1.39	5.64 ± 0.56	7.26 ± 1.47	6.26 ± 1.03	8.55 ± 0.62
	NW	3.58 ± 0.73	**4.55 ± 1.36**	7.44 ± 0.44	5.65 ± 0.52	7.45 ± 0.43	6.40 ± 0.40	6.67 ± 0.77	6.33 ± 0.62	7.44 ± 0.95	6.32 ± 0.74	8.68 ± 0.39
GOS	MOB	4.93 ± 1.44	**3.00 ± 0.00***	**8.17 ± 0.55***	5.81 ± 0.46	7.84 ± 0.52	7.13 ± 1.03	6.69 ± 1.64	6.62 ± 0.55	8.12 ± 0.66	6.49 ± 1.03	8.95 ± 0.41
	NW	3.81 ± 1.00	**4.66 ± 1.38**	**7.48 ± 0.56**	5.75 ± 0.42	7.82 ± 0.58	6.75 ± 0.33	6.97 ± 0.86	6.92 ± 0.57	7.82 ± 0.94	6.79 ± 0.40	8.70 ± 0.33
Inulin	MOB	4.50 ± 1.01	**3.15 ± 0.29***	7.12 ± 1.01	5.19 ± 0.82	6.79 ± 0.92	5.71 ± 0.54	**5.85 ± 1.04***	5.43 ± 0.82	7.44 ± 1.29	6.32 ± 1.10	8.48 ± 0.69
	NW	3.66 ± 0.82	**4.58 ± 1.38**	7.21 ± 0.38	5.73 ± 0.73	7.40 ± 0.60	6.27 ± 0.62	**7.01 ± 0.59**	5.85 ± 0.87	8.01 ± 0.54	6.84 ± 0.52	8.63 ± 0.44
P95	MOB	4.87 ± 1.46	**3.00 ± 0.00***	7.99 ± 0.58	5.45 ± 0.38	7.04 ± 0.88	6.49 ± 1.36	6.17 ± 1.41	5.50 ± 0.68	8.12 ± 0.34	6.26 ± 1.34	8.88 ± 0.28
	NW	3.90 ± 1.02	**4.62 ± 1.33**	7.50 ± 0.47	5.73 ± 0.33	7.39 ± 0.52	6.30 ± 0.45	7.25 ± 0.88	6.39 ± 0.61	7.90 ± 0.93	6.84 ± 0.37	8.76 ± 0.24
Synergy1	MOB	4.91 ± 1.28	**3.21 ± 0.39***	7.58 ± 0.82	5.28 ± 0.91	6.98 ± 0.76	6.04 ± 0.81	6.15 ± 1.39	5.67 ± 0.62	7.34 ± 1.40	6.34 ± 1.18	8.56 ± 0.69
	NW	3.75 ± 0.99	**4.58 ± 1.40**	7.20 ± 0.44	5.46 ± 0.55	7.41 ± 0.56	6.27 ± 0.51	7.48 ± 1.31	6.23 ± 0.68	7.91 ± 0.86	6.63 ± 0.50	8.60 ± 0.36

Table S3. Medium and interquartile range of relative *Bifidobacterium* species assessed by ITS-region sequencing at time 0, before the faecal cultures in NW and MOB microbiota. Bifidobacterial species were filtered by minimum abundance of 1%. "*" shows differences between both population groups (p-value <0.05).

ITS-sequecing	MOB	NW
Richness	12.50 [12.00 - 13.00]*	10.00 [8.00 - 11.00]
B. adolescentis	42.79 [31.21 - 59.79]*	28.76 [7.77 - 79.96]
B. animalis subsp *lactis*	1.07 [0.45 - 1.39]*	0.39 [0.10 - 1.08]
B. bifidum	1.25 [0.76 - 1.66]	0.53 [0.05 - 3.61]
B. breve	3.30 [1.97 - 4.50]*	0.29 [0.03 - 0.59]
B. catenulatum	1.15 [0.61 - 2.73]*	0.06 [0.00 - 3.15]
B. choerinum	0.50 [0.26 - 1.44]*	0.00 [0.00 - 0.53]
B. crudilactis	2.12 [0.82 - 3.03]*	15.54 [2.90 - 38.77]
B. dentium	3.20 [2.10 - 3.81]*	0.48 [0.06 - 3.60]
B. longum subsp *longum*	22.29 [15.27 - 24.92]*	12.72 [6.91 - 46.09]
B. mongoliense	0.11 [0.06 - 0.31]*	1.85 [0.30 - 5.28]
B. pseudocatenulatum	6.90 [2.95 - 12.98]*	1.32 [0.22 - 2.45]
Others	6.63 [2.67 - 9.78]	2.32 [0.29 - 3.88]

Table S4. Statistical tests carried out studying each variable independly in each population group. K-W means Kruskal-Wallis test

Analysis	Variable	MOB	NW	Comparision between populations
16S rRNA gene profiling	All taxonomic ranks	K-W	K-W	MANN WHITNEY U
Bifidobacterial ITS	All species	K-W	K-W	MANN WHITNEY U
Microbiota composition by qPCR	*Lactobacillus* group	K-W	K-W	MANN WHITNEY U
	Akkermansia	K-W	K-W	MANN WHITNEY U
	Bacteroides group	K-W	K-W	MANN WHITNEY U
	Faecalibacterium spp.	ANOVA	ANOVA	T-STUDENT
	Bifidobacteirum spp.	ANOVA	ANOVA	T-STUDENT
	Enterobacteria	K-W	K-W	MANN WHITNEY U
	Clostridium cluster XIVa	K-W	ANOVA	MANN WHITNEY U
	Total bacteria	K-W	ANOVA	MANN WHITNEY U
	B. longum	K-W	ANOVA	MANN WHITNEY U
	B. catenulatum	ANOVA	ANOVA	T-STUDENT
	B. adolescentis	ANOVA	ANOVA	T-STUDENT
Gas	Cumulative gas	ANOVA	ANOVA	T-STUDENT
pH-Increments respect to time 0	ΔpH	K-W	K-W	MANN WHITNEY U
SCFA-Increments respect to time 0	Acetate	K-W	K-W	MANN WHITNEY U
	Propionate	ANOVA	K-W	MANN WHITNEY U
	Butyrate	K-W	K-W	MANN WHITNEY U
	BSCFA	K-W	K-W	MANN WHITNEY U
	Total SCFA	K-W	K-W	MANN WHITNEY U
Interaction with HT29-AUC	FS	K-W	K-W	MANN WHITNEY U
	IM	K-W	K-W	MANN WHITNEY U
